# Meteorin-β: A Novel Biomarker and Therapeutic Target on Its Way to the Regulation of Human Diseases

**DOI:** 10.3390/ijms26104485

**Published:** 2025-05-08

**Authors:** Bei Wang, Xiao Li, Xun Gao

**Affiliations:** 1 Center of Clinical Laboratory Medicine, Zhongda Hospital, Southeast University, Nanjing 210009, China; wb671228@163.com (B.W.); 19993954612@163.com (X.L.); 2 Department of Laboratory Medicine, Medical School of Southeast University, Nanjing 210009, China

**Keywords:** Metrnβ, biological functions, diagnosis, therapeutics, human diseases

## Abstract

The novel secreted protein Meteorin-β (Metrnβ) is a homologous protein of the neurotrophic regulator Meteorin, which is widely expressed in the skin, mucous membranes, and white adipose tissue upon stimulation by a variety of inflammatory mediators, including cytokines and chemokines, while, at the same time Metrnβ may also regulate the expression of these cytokines and chemokines. As a small secreted protein with low tissue specificity, Metrnβ plays vital roles in energy metabolism, insulin sensitivity regulation, neurodevelopment, white fat browning, and inflammatory response. Specifically, Metrnβ may act as an adipokine, myokine, neurotrophic factor, and cytokine, thereby being involved in the pathological and physiological processes of various human diseases, including metabolic, autoimmune and infectious/allergic diseases, and certain types of tumors. This review aims to systematically introduce the current research progress on Metrnβ, including its expression and distribution profiles, biological functions, and immunomodulatory roles in the process of human diseases. Additionally, we also discuss its potential as a biomarker, as well as a therapeutic/preventive agent for human diseases.

## 1. Introduction

Since the identification and discovery of Meteorin-like protein (Metrnl/Metrnβ), also designated as interleukin-41 (IL-41), in 2006 [1], a plethora of studies have focused on its emerging role as a secretory protein implicated in diverse pathophysiological processes. As a potent immunomodulator, Metrnβ exhibits inflammation-associated expression dynamics that critically regulates disease progression. Metrnβ is highly expressed in adipocytes, endothelial cells, activated monocytes, and myocytes of barrier tissues (e.g., skin, white adipose tissue, and mucosal membranes of the digestive and respiratory tracts) upon stimulation. In contrast, its expression in the central nervous system (CNS) is comparatively lower [2,3]. The functional role of secreted protein like Metrnβ always relies on its binding to the specific receptor. In 2024, Reboll et al. identified for the first time the receptor tyrosine kinase (KIT) on endothelial cells as a high-affinity receptor for Metrnβ but the specific receptor of Metrnβ is currently unclear [4]. Despite this, mechanistic studies have revealed its pivotal roles as an immunomodulator. Indeed, Metrnβ deficiency exacerbates inflammatory pathogenesis, as evidenced by Metrnβ^−/−^ mice displaying reduced serum IgG, systemic inflammatory lesions, and elevated mortality [3]. Furthermore, accumulating evidence reveals its pleiotropic functions, including modulation of inflammatory cascades [2,5,6,7], promotion of angiogenesis and tissue remodeling, enhancement of musculoskeletal repair neurite outgrowth, and regulation of metabolic homeostasis via white adipose browning, and insulin sensitivity improvement [2,7,8,9,10,11,12,13]. These multifunctional properties position Metrnβ as both a promising diagnostic biomarker and a therapeutic target for numerous clinical disorders, particularly metabolic diseases like diabetes and obesity, cardiovascular pathologies, and inflammatory conditions involving autoimmunity [14,15,16,17]. Notably, in allergy, we have previously demonstrated its capacity to attenuate the progress of allergic asthma and atopic dermatitis (AD) [18,19]. Moreover, our clinical observations demonstrated elevated Metrnβ levels correlating with coronavirus disease 2019 (COVID-19) severity, suggesting its involvement in SARS-CoV-2 pathogenesis [20]. Recent studies have also underscored its pivotal role in malignancies [21,22,23]. Whereas previous reviews have summarized its functions and regulations in cardiometabolic as well as autoimmune diseases, the novelty of our review is that we systematically discuss the biosynthesis, functional mechanisms, and therapeutic potential of Metrnβ across human disease spectra, involving those recently focused on, such as cancer and infectious diseases, which would help provide guidance for its values as a diagnostic, prognostic, and therapeutic target in human diseases.

## 2. The Discovery and Structure of Metrnβ

Metrnβ was first identified in 2006 through the Body Index of Gene Expression (BIGE) database [1]. The gene encoding Metrnβ, referred to as C17ORF99, consists of four exons that code for 311 amino acids across 936 base pairs. Post-translational cleavage of the 45-residue N-terminal signal peptide generates a mature 266-amino acid protein (27 kDa) lacking transmembrane domains, namely Metrnβ [12,24]. According to the NCBI database, C17ORF99 is located on the qE2 locus of chromosome 11 in mice and q25.3 on chromosome 17 in humans, with 77% amino acid conservation between these species (239/311 residues) [9,13]. Phylogenetic analyses reveal homologous genes for Metrnβ across vertebrates (zebrafish, *Xenopus*), but none in invertebrates such as *Drosophila melanogaster* or *Caenorhabditis elegans* [13,25]. Intriguingly, two paralogs (*CiMetrnl-a* and *CiMetrnl-b*) were identified in grass carp (*Ctenopharyngodon idella*), with *CiMetrnl-a* constitutively expressed in healthy tissues, while *CiMetrnl-b* remains undetectable, suggesting potential regulatory divergence of these proteins. Notably, current studies found that Metrnβ shares 40% homology with Meteorin, as it has a 46% amino acid sequence similarity to Meteorin, and together, these proteins form a novel protein family, albeit with distinct functions, requiring further investigation [13,24,25,26,27,28].

## 3. Distribution and Expression Patterns of Metrnβ

As a low-specificity secretory protein, Metrnβ exhibits broad systemic expression with predominant localization in barrier tissues such as skin, mucosa, and adipose and skeletal muscle tissue after cold exposure and exercise [7,13,24]. In adipose tissue, its expression is markedly higher in subcutaneous white adipose tissue than in brown adipose tissue of both rodents and humans [24]. Further immunohistochemical analyses reveal diffuse Metrnβ distribution throughout adipose tissue, excluding lipid droplets, without significant expression differences between adipocytes and stromal cells [24]. Metrnβ is also detectable in muscle, liver, spleen, heart, and the CNS [13,24]. Notably, in the murine CNS, Metrnβ displayed dynamic spatiotemporal expression patterns, with initial expression in the embryonic neural tube floor (E9.5), weak inner ear expression at E13.5 followed by sharp upregulation at E14.5, and robust embryonic cartilage expression post-E14.5. However, adult mice exhibited negligible neural Metrnβ expressions [13]. Metrnβ also shows abundant expression in human cerebral blood vessels, yet its levels in parenchymal brain expression remain sparse [29]. As far as the cellular sources are concerned, Metrnβ is produced by bone marrow-derived macrophages upon stimulation, as well as by resting fibroblasts and keratinocytes treated with IFN-γ, and Metrnβ in alternatively activated macrophages may also be released in response to Th2 cytokines IL-4 and IL-13 [3,7]. However, it should be noted that synthesis of Metrnβ is context-dependent, modulated by physiological and pathological factors like temperature, exercise, obesity, and pharmacological interventions [2,24].

## 4. Biological Functions of Metrnβ

**Figure 1:**
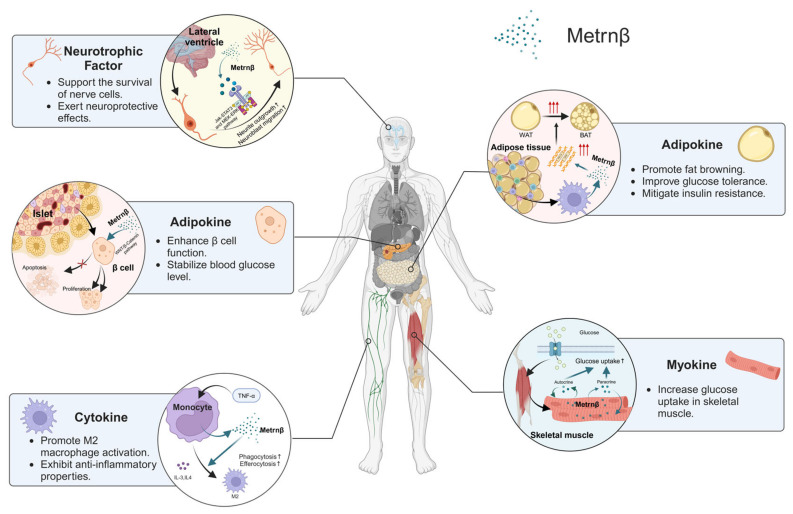
The biological functions of Metrnβ. Initially, Metrnβ mainly served as an adipokine to promote fat browning, improve glucose tolerance, and mitigate insulin resistance. When working as a neurotrophic factor, Metrnβ may support the survival of nerve cells, therefore exerting neuroprotective effects. As a myokine, Metrnβ may increase the uptake of glucose in skeletal muscles. Additionally, Metrnβ is a novel anti-inflammatory cytokine with pivotal roles in promoting M2 macrophage polarization and suppressing the expression of inflammatory mediators. These multifunctional properties enable Metrnβ to serve as a novel immune regulator in human diseases. The arrows in the figure indicate promotion/increase/upregulation.

Since the discovery of Metrnβ, researchers have highlighted it as a multifunctional mediator with regulatory roles as an adipokine and myokine. Later, more and more studies gradually focused on its roles as a cytokine, and also as a neurotrophic factor, exerting protective effects in metabolic regulation, neurodevelopment, and inflammatory diseases [15,16,30] (Figure 1).

### 4.1. Regulation of Lipid Metabolism and Insulin Resistance as an Adipokine

Originally classified as an adipokine, Metrnβ has attracted substantial research interests due to its multifaceted therapeutic potential in metabolic regulation, possibly via maintaining glucose homeostasis, augmenting insulin responsiveness, inducing adipose tissue browning, and promoting energy expenditure. Specifically, Metrnβ expression decreases in white adipose tissue under normal conditions but increases during high-fat diet (HFD)-induced obesity and adipocyte differentiation in mice [24]. By using models of both HFD-fed Metrnβ knockout mice and Metrnβ transgenic overexpression mice, Metrnβ has been proved to enhance adipocyte differentiation by upregulating peroxisome proliferator-activated receptor gamma (PPARγ) and C/EBPα, while reducing lipid accumulation via regulating lipogenesis-related genes like ACC and FASN [9]. Moreover, Metrnβ improves systemic glucose homeostasis of mice by stimulating thermogenic genes peroxisome proliferator-activated receptor gamma coactivator 1-alpha (PGC-1α) and UCPs [2,31], activating WNT/β-Catenin to preserve β-cell function [32], and alleviating insulin resistance through AMPK/PPAR-mediated fatty acid oxidation and anti-inflammatory effects [6,8]. These findings collectively position Metrnβ as a multifunctional adipokine coordinating metabolic homeostasis and tissue repair. However, its interactions with canonical adipokines such as leptin, adiponectin, and visfatin remain underexplored. Intriguingly, Metrnβ exhibits functional convergence with adiponectin in enhancing insulin sensitivity and mitochondrial biogenesis through shared AMPK-dependent pathways [33]. Although no direct evidence links Metrnβ to leptin signaling, both adipokines demonstrate parallel metabolic effects, as leptin activates POMC neurons to drive adipose browning [34,35], suggesting their potential regulatory interplay in adipose remodeling. Future studies should delineate whether Metrnβ functionally complements or antagonizes established adipokine networks through shared transcriptional regulators or compensatory feedback loops.

### 4.2. Support of Neuronal Development as a Neurotrophic Factor

Several secreted proteins, such as nerve growth factor (NGF), brain-derived neurotrophic factor (BDNF), and Meteorin as well as Metrnβ, are well-established neurotrophic factors that support nerve cells and play a central role in the growth, development, and functional integrity of neurons [36]. Among them, Metrnβ was shown to promote hippocampal neurite extension and sensory neuron growth as a neurotrophic factor [37]. It was demonstrated that the use of siRNAs targeting Metrnβ could significantly inhibit neurite elongation in rat, an effect that was partially rescued by Metrnβ, highlighting its indispensable role in this process [37]. Similarly, it was found that Metrnβ induced neurite outgrowth in sensory neurons in vitro through the Jak/STAT3 and MEK/ERK pathways. Furthermore, it significantly enhances neuroblast migration within the subventricular zone (SVZ) while promoting the survival of spiral ganglion neurons [13]. Moreover, Metrnβ may attenuate age-induced cognitive dysfunction via regulating the levels of neurotrophic factors such as BDNF, tropomyosin receptor kinase B (TrkB), and glial fibrillary acidic protein (GFAP) in D-galactose (D-gal)-induced aging mice [38]. Depletion of Metrnβ is associated with mild 17q12 syndrome, characterized by intellectual disability and growth retardation [39]. Collectively, these data underscore its neuroprotective potential in neurodegenerative disorders.

### 4.3. Contribution to Muscle Regeneration and Repair as a Myokine

Metrnβ is synthesized and secreted by skeletal muscle and adipose tissue during exercise or cold exposure, where it suppresses NLRP3 inflammasome activation and IL-1β/IL-18 expression. This action mitigates chronic inflammation that would otherwise impede muscle recovery [2,40]. As a secreted factor induced by exercise, Metrnβ enhances glucose uptake and fatty acid oxidation in skeletal muscle, thereby providing essential energy substrates for tissue repair and regeneration. Furthermore, Metrnβ mediates interorgan communication between skeletal muscle and adipose tissue, coordinating systemic metabolic adaptations that promote muscle regeneration [41]. Mechanistically, in skeletal muscle regeneration, Metrnβ attenuates lipid-induced inflammation and insulin resistance through AMPK/PPARδ pathway activation in both palmitate-treated differentiated C2C12 cells and the skeletal muscle of HFD-fed mice. This results in downregulation of multiple inflammatory mediators, including NF-κB nuclear translocation, IκBα phosphorylation, and expression of IL-6, TNFα, and CCL2, etc. [8]. Concurrently, Metrnβ drives macrophage polarization toward the anti-inflammatory M2 phenotype via Stat3 activation, and stimulates macrophage-derived IGF-1 production. These paracrine effects directly enhance primary muscle satellite cell proliferation and myogenesis [42]. Notably, Metrnβ also exerts autocrine/paracrine effects to enhance glucose uptake and fatty acid oxidation, further facilitating muscle regeneration [43].

### 4.4. Anti-Inflammatory Cytokine

Metrnβ exhibits context-dependent immunoregulatory functions with demonstrated anti-inflammatory predominance across multiple pathological conditions. In several pathological conditions, Metrnβ was shown to be widely expressed in cytokine-activated macrophages, especially by TNF-α, while IFN-γ/TGF-β suppress its synthesis [3,7]. Metrnβ^−/−^ mice exhibit increased susceptibility to inflammatory lesions and elevated mortality [3], indicating that Metrnβ is a potential candidate with dual roles in regulating inflammation. Mechanistically, Metrnβ orchestrates multi-modal anti-inflammatory effects through downregulating TNF-α/MCP-1 expression [5], activating AMPK-PAK2 to mitigate cardiomyocyte stress [44], and promoting M2 macrophage polarization [2], collectively contributing to its therapeutic potential in coronary artery disease and related inflammatory disorders. Intriguingly, while Metrnβ enhances type 2 immunity via eosinophil-mediated IL-4/IL-13 elevation in adipose tissue [2], it conversely suppresses Th2 responses during allergy [18,19]. This dichotomy extends to Th1 immunity, where Metrnβ inhibits canonical Th1 pathways [7,26,45], positioning it as a broad-spectrum anti-inflammatory mediator. Nevertheless, several pieces of evidence identify Metrnβ as a pleiotropic cytokine capable of context-dependent proinflammatory actions. For instance, in specific inflammatory milieus, Metrnβ directly activates the NF-κB pathway, augmenting proinflammatory cytokines (IL-1β, IL-6, IL-8, TNF-α) in leukocytes [28], and amplifies inflammatory responses to bacterial challenge [46]. Such context-dependent functional switching likely originates from tissue-specific receptor isoform engagement, dynamic post-translational modifications, and spatiotemporal regulation of Metrnβ’s bioavailability.

## 5. Involvement of Metrnβ in Human Diseases

**Figure 2:**
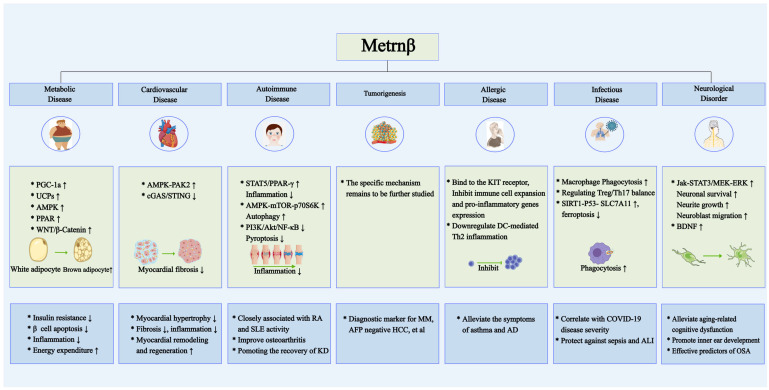
Potential regulatory mechanism of Metrnβ in human diseases as a novel immunoregulator. In metabolic and cardiovascular diseases, Metrnβ may work as a pivotal regulator. Metrnβ also participated in the pathophysiology of autoimmune, allergic, and infectious diseases like SLE, asthma, and sepsis. Additionally, Metrnβ regulates neurological disorders such as inner ear development, and may be correlated with disease progression in malignances. The arrows ↑ and → in the figure indicate promotion/increase/upregulation, and ↓ indicate inhibition/decrease/downregulation. The * indicates serial number.

Based on these vital biological functions, expression and secretion of Metrnβ fluctuate in response to pathophysiological changes in vivo. In the past 20 years since its discovery, accumulating evidence has revealed that distinct Metrnβ expression patterns under pathological conditions are closely associated with the initiation and progression of a broad spectrum of human diseases. These range from extensively studied metabolic, autoimmune, and cardiovascular disorders to emerging research areas such as allergic and infectious diseases (Table 1). In this review, we systematically summarize the dual roles and molecular mechanisms of Metrnβ in human diseases based on its pivotal biological functions described above (Figure 2).

### 5.1. Metrnβ in Metabolic Disease: Interplay Between Diabetes and Obesity

Over the decades, clinical findings on circulating Metrnβ levels in type 2 diabetes (T2D) remain contradictory, with studies reporting decreased [47,48,49,50], elevated [51,52,53,54,55,56], or unchanged levels compared to non-diabetic controls [57,58]. Such discrepancies may stem from confounding factors including pharmacological interventions, disease progression, and heterogeneity in age, BMI, and metabolic status [65]. Notably, Metrnβ also exhibits therapeutic potential in diabetic complications, accelerating neovascularization and wound healing in diabetic ulcers [10]. Intriguingly, Metrnβ’s metabolic influence extends to obesity-associated pathophysiology. While it enhances thermogenesis and adipocyte differentiation [59,60], clinical correlations between Metrnβ and obesity also display paradoxical trends. Elevated circulating Metrnβ levels have been observed in obese individuals [2,24,53,60,61], contrasting with reports of reduced [51,62] or unchanged levels [6,47,48,56,63]. This inconsistency parallels findings in T2D, suggesting shared regulatory complexity. Notably, weight loss interventions reveal transient dynamics of Metrnβ: bariatric surgery and caloric restriction induce a short-term Metrnβ surge at 3 months, followed by normalization at 6–12 months, implying a compensatory adaptation to metabolic stress [59,64]. These fluctuations correlate with dual metabolic benefits, improved insulin sensitivity [6,62] and lipid homeostasis [6,48,51,52,65,66,67], highlighting Metrnβ’s role as a metabolic modulator at the diabetes–obesity nexus. Together, the current evidence underscores the need for longitudinal studies with rigorous phenotyping to dissect Metrnβ’s context-dependent roles in T2D and obesity. Future research should prioritize standardization of confounding variables such as medication use and adiposity distribution and exploration of tissue-specific Metrnβ actions to reconcile existing paradoxes in both T2D and obesity.

### 5.2. Metrnβ in Cardiovascular Pathophysiology and Therapeutic Potential

Clinical and experimental evidence positions Metrnβ as a pivotal regulator in cardiovascular homeostasis, with dual roles spanning from endothelial protection to myocardial repair. In atherosclerosis, an inverse correlation was observed between circulating Metrnβ levels and endothelial dysfunction, demonstrating that hypo-metrnβ predicts impaired flow-mediated dilation in human [47]. This observation is corroborated by murine models showing 62% reduction in aortic Metrnβ expression during atherogenesis, accompanied by diminished eNOS Ser1177 phosphorylation and nitric oxide bioavailability [29]. Clinically, it was discovered that serum Metrnβ levels were significantly lower in patients with coronary heart disease (CHD) compared to controls, and individuals with lower Metrnβ levels had a 1.5-fold higher risk of developing CHD than those with higher levels [65,68]. Additionally, Metrnβ levels were found to be decreased in patients with acute coronary syndrome (ACS) [63], highlighting its potential value in distinguishing ACS patients from healthy controls.

The cardioprotective repertoire of Metrnβ extends to myocardial remodeling and regeneration. In a model of isoproterenol-induced cardiac hypertrophy and fibrosis, Metrnβ^−/−^ mice exhibited more severe heart damage, mediated through the PPARα pathway [69]. It was observed that patients with cardiac dysfunction had lower serum levels of Metrnβ, accompanied with weight loss and greater severity of cardiac dysfunction [70]. Similarly, there was a reduction in Metrnβ expression in the rodent heart following doxorubicin-induced toxic injury, where Metrnβ knockdown exacerbated doxorubicin-induced cardiotoxicity and increased mortality [71]. Mechanistically, restoration of Metrnβ signaling via adenoviral delivery attenuates fibrotic remodeling while activating cardiomyocyte proliferative programs in mice [69,71]. Intriguingly, Metrnβ demonstrates dual receptor specificity, binding KIT on endothelial cells to stimulate angiogenesis while activating cardiomyocyte SIRT1 via paracrine cAMP/PKA signaling, thereby reducing doxorubicin-induced apoptosis [4,71]. Moreover, heart-specific overexpression of Metrnβ significantly improved hypertension and mitigated pathological cardiac hypertrophy in hypertensive rats possibly by inhibiting Ang-II-induced autophagy of cardiomyocytes via regulation of the BRCA2/Akt/mTOR signaling pathway [72]. Notably, the molecular mechanism underlying Metrnβ’s cardioprotective effects in cardiomyopathy also involves the inhibition of the LKB1/AMPK/ULK1-mediated autophagy-dependent cGAS/STING signaling pathway, as well as the attenuation of endoplasmic reticulum stress and the diminish of cardiomyocyte apoptosis via the AMPK/PAK2 pathway [44,73]. Most recently, it was also found that Metrnβ ameliorated myocardial ischemia-reperfusion injury by activating AMPK-mediated M2 macrophage polarization both in vitro and in vivo [74]. Collectively, these findings highlight the significant cardioprotective role of Metrnβ and suggest that it may serve as a novel cardiac factor critical for maintaining the physiological microenvironment of the heart and protecting cardiomyocytes.

### 5.3. Metrnβ in Autoimmune Disorders: From Pathogenesis to Biomarker Potential 

Previous studies have summarized the dual immunoregulatory roles of Metrnβ across autoimmune disorders. In patients with rheumatoid arthritis (RA), elevated synovial and serum Metrnβ levels were observed and correlated with disease activity, suggesting its involvement in synovial inflammation [7,87]. However, in Graves’ disease (GD), paradoxical findings were found. Some studies suggest that hyperthyroidism-associated elevation of Metrnβ may regulate lipid metabolism through the STAT5/PPAR-γ pathway [6]. In contrast, other research reports reduced serum Metrnβ level correlating with systemic inflammation in GD patients [88]. This inconsistency may reflect Metrnβ’s involvement in modulating M1/M2 macrophage balance, requiring further mechanistic studies. Psoriatic arthritis (PsA) patients exhibit synovial-specific Metrnβ overexpression, driven by TNF/IL-17A synergy in stromal cells [7,26,27], though its functional effect remains undefined. In patients with Crohn’s disease (CD), lower circulating levels of Metrnβ with an inverse correlation between its levels and BMI, IL-6, and TNF-α concentrations was observed [89], while mesenteric adipose Metrnβ was observed to be upregulated and be beneficial to CD, partly via mediating STAT5-dependent anti-inflammatory responses to reduce disease activity [45] and enhanced autophagy in epithelial cells [90]. Recent studies have highlighted the importance of Metrnβ in the pathophysiology of osteoarthritis (OA), establishing a significant association of Metrnβ with its onset and progression. Metrnβ exhibits dual compartmentalization in OA patients, with reduced serum levels correlating with disease severity, yet elevated synovial concentrations linked to insulin resistance, contrasting with its known insulin-sensitizing effects in metabolic disorders [91]. Mechanistically, Metrnβ protects rat chondrocytes from inflammation and pyroptosis via suppressing PI3K/Akt/NF-κB and blocking NLRP3/caspase-1/GSDMD activation [92]. Concurrently, it enhances PPARγ/PPARδ-mediated autophagic protection to mitigate cartilage degradation, positioning it as a multi-target therapeutic candidate for OA [5,6,8,93]. These findings underscore Metrnβ’s context-dependent functionality, warranting mechanistic studies to clarify its therapeutic potential across autoimmune spectra. Notably, very recent studies have also highlighted the regulatory roles of Metrnβ in other autoimmune diseases that have not been summarized before.

#### 5.3.1. Metrnβ in Systemic Lupus Erythematosus (SLE)

SLE is a debilitating autoimmune disease with significant morbidity and mortality with pathogenesis involving interactions between various immune cells and inflammatory mediators [94,95]. Recently, we observed for the first time a substantial increase in circulating Metrnβ levels in SLE patients, with concentrations being higher in active patients compared to those in remission. Additionally, Metrnβ expression was positively correlated with clinical indicators of disease activity and negatively correlated with regulatory T cells (Tregs), B10 cells, and innate lymphoid cells (ILCs), suggesting that Metrnβ is involved in the onset and progression of SLE and may serve as a useful biomarker for distinguishing SLE patients from healthy individuals [96]. Targeting Metrnβ with specific therapies could therefore hold promise as a novel strategy for improving disease outcomes in SLE.

#### 5.3.2. Metrnβ in Kawasaki Disease (KD)

KD, an acute systemic vasculitis predominantly affecting young children, remains a leading cause of acquired coronary artery abnormalities in developed nations [97].While intravenous immunoglobulin (IVIG) therapy significantly reduces coronary artery lesion (CAL) incidence, approximately 10–20% of patients demonstrate treatment resistance, necessitating improved predictive biomarkers for IVIG non-responsiveness and CAL development [98]. Recent breakthroughs identify Metrnβ as a key regulator in KD pathogenesis. Clinical investigations demonstrate significantly elevated circulating Metrnβ levels in acute-phase KD patients compared to age-matched controls and an inverse correlation emerges between Metrnβ and IgM titers [99]. As immunoglobulin levels (particularly IgM) correlate with favorable KD outcomes [100], this paradoxical relationship implies potential regulatory cross-talk, where Metrnβ might modulate post-acute phase inflammation through IgM-dependent pathways. Strikingly, multivariate analyses revealed independent correlations between plasma Metrnβ concentrations and D-dimer and NT-proBNP [99], both of which are established risk factors for CAL in KD [101]. Additionally, serum Metrnβ levels are markedly increased in IVIG-resistant and CAL groups, with a positive correlation with inflammatory markers including erythrocyte sedimentation rate (ESR), C-reactive protein (CRP), and CRP/albumin ratio [102], indicating that Metrnβ may play a significant role in the pathogenesis of coronary artery complications in KD. Mechanistically, Metrnβ’s anti-inflammatory effects on macrophage polarization and endothelial protection [2,3,7,103] could possibly mediate vascular repair during convalescence of KD. However, current clinical evidence remains observational, necessitating validation through multicenter prospective cohorts and mechanistic studies to determine whether Metrnβ elevation represents a compensatory protective response or directly contributes to CAL pathogenesis. Moreover, future research should also prioritize longitudinal assessments of Metrnβ kinetics, tissue-specific expression profiling, and integration with established risk scores to evaluate its potential as a biomarker for personalized IVIG therapy optimization.

#### 5.3.3. Metrnβ in Spondylitis

Ankylosing spondylitis (AxSpA), a prototypical inflammatory spondyloarthropathy, manifests through progressive spinal ankylosis and sacroiliac joint erosion, with chronic back pain and functional impairment constituting its pathognomonic features [104]. In 2023, Uçar IMB, et al., reported for the first time lower serum concentrations of Metrnβ in patients with AxSpA compared to healthy controls, paralleling reductions in canonical inflammatory mediators such as IL-6, IL-17A, and TNF-α [105]. However, the underlying mechanisms driving this phenomenon remain unclear. Notably, most patients in these studies were undergoing biological therapy, suggesting that the observed reductions in serum Metrnβ levels and inflammatory markers may be influenced by their treatment regimens. Contrastingly, a recent longitudinal cohort study employing treatment-naïve AxSpA patients demonstrated elevated baseline serum Metrnβ levels that positively correlated with ASDAS-CRP scores and MRI-detected spinal inflammation, and exhibited strong diagnostic validity for AxSpA [106]. This dichotomous expression pattern implies phase-specific regulatory roles of Metrnβ in AxSpA: suppressed Metrnβ during biologic therapy may reflect successful inflammatory control, whereas elevated levels in active disease suggest compensatory immunomodulation. Together, these findings support the potential roles of Metrnβ as a novel biomarker for diagnosing AxSpA, stratifying disease severity, and monitoring therapeutic response. Such insights could transform the diagnosis and management of AxSpA, offering new avenues for improving patient care and prognosis.

### 5.4. Metrnβ in Human Malignancies: Oncogenic Implications

#### 5.4.1. Metrnβ in Breast Carcinogenesis

The expression of Metrnβ has been observed in invasive ductal breast cancer, which showed that Metrnβ levels were elevated in breast cancer patients across grades 1, 2, and 3 compared to healthy controls. However, no significant differences in Metrnβ expression were detected among different stages of breast cancer tissue [22]. Similar findings were reported by Kocaman et al. [75], indicating that while Metrnβ may be associated with the presence of breast cancer, its expression does not appear to vary significantly with tumor progression. However, the detailed regulatory mechanisms with regard to Metrnβ in breast cancer warrants future in depth studies. Metrnβ’s role in STAT3/NF-κB crosstalk, activating STAT3 while suppressing NF-κB-driven proinflammatory cytokines to create an immunosuppressive niche [42], may possibly explain this dichotomy; future organoid models with inducible Metrnβ expression could clarify its context-dependent roles.

#### 5.4.2. Metrnβ in Cutaneous Neoplasms

Several studies have reported a statistically significant elevation of Metrnβ levels in both basal cell carcinoma and trichoblastoma, suggesting that Metrnβ may serve as a distinguishing marker between these two conditions [21,76]. However, it is worth noting that Ushach et al. did not observe significant change with regard of skin Metrnβ expression in basal cell carcinoma patients [7]. This discrepancy may be due to limited sample sizes or varying degrees of disease activity in the studied cohorts. Further large-scale research is needed to clarify the expression patterns and mechanisms of Metrnβ in basal cell carcinoma and to validate its potential as a diagnostic marker. However, the role of Metrnβ in cutaneous neoplasm development is not fully understood. Metrnβ may play a key role in tumor formation due to its involvement in the regulation of angiogenesis [107].

#### 5.4.3. Metrnβ in Malignant Mesothelioma (MM)

A retrospective study on MM found that Metrnβ exhibited higher immunoreactivity in MM compared to reactive mesothelial hyperplasia (RMH), suggesting that Metrnβ could potentially serve as a marker to distinguish MM from mesothelial hyperplasia [77]. Since cell proliferation in MM is closely linked to increased energy expenditure associated with glucose metabolism, and the greater the malignancy of the tumor, the higher the energy demands, it is therefore possible that the stronger expression of Metrnβ in MM may represent a compensatory anti-tumor response by the host based on the pleiotropic effects of Metrnβ in improving insulin sensitivity, maintaining glucose homeostasis, promoting white fat browning, and increasing energy expenditure [9]. However, due to the small sample size of this study, further large-scale studies are necessary to investigate the expression and mechanism of Metrnβ in malignant mesothelioma.

#### 5.4.4. Metrnβ in Ovarian Tumorigenesis

Metrnβ is a protein with potential relevance to various ovarian health conditions, particularly in the context of Polycystic Ovary Syndrome (PCOS) and recurrent pregnancy loss (RPL), where serum levels of Metrnβ are lower in women with PCOS and RPL compared to healthy controls [108]. Ovarian cancer is prevalent among women with its pathogenesis involving genetic factors and environmental and lifestyle influences. In 2023, it was demonstrated for the first time that Metrnβ was primarily localized in the epithelial regions of normal ovarian tissue, whereas in cancerous tissues, Metrnβ immunoreactivity was observed in the parenchymal areas, and Metrnβ levels were found to be highest in the control group, diminished in benign tumors, and lowest in malignant tumors [78]. These findings suggest that reduced Metrnβ expression may be linked to the progression of and may also serve as a potential diagnostic biomarker for ovarian cancer.

#### 5.4.5. Metrnβ in Colorectal Cancer (CRC): Dual Oncogenic Paradigm

Specifically, Metrnβ expression levels are elevated in CRC tissues compared to healthy tissues, particularly in advanced stages, which is associated with poor prognosis in CRC patients [80]. Conversely, a 2023 multi-center analysis reported 40% lower Metrnβ immunoreactivity in primary tumors versus healthy mucosa, particularly in microsatellite instability-high (MSI-H) subtypes [81]. This discrepancy may arise from tumor microenvironment heterogeneity or technical variability in antibody validation. However, no mechanism studies have investigated the detailed regulatory functions of Metrnβ in CRC. In 2024, by functional genomics studies employing CRISPR-Cas9, Metrnβ was identified as a susceptibility gene alongside TRPS1 and C14orf166 for CRC, demonstrating oncogenic properties through enhanced cell proliferation and migration in CRC cell lines [79]. Notably, Metrnβ decouples the electron transport chain in CD8^+^ T cell mitochondria via E2F-PPARδ to disrupt CD8^+^ TIL mitochondrial function and inhibit its anti-tumor activity in CRC [109]. While these in vitro findings regarding Metrnβ in CRC provide mechanistic insights into CRC etiology, the absence of in vivo validation and comprehensive pathway mapping limits clinical translation. Given that Metrnβ functions as both an adipokine and an endothelial modulator, and that preclinical models demonstrate that it can both activate PI3K/AKT/mTOR signaling and suppress pro-apoptotic BAX expression to promote tumor cell survival, as well as that a lack of Metrnβ in endothelial cells disrupts vascular homeostasis in colorectal niches [47,110], it is possible that Metrnβ regulates endothelial cell function in the colorectum of colorectal cancer. However, this possibility has yet to be thoroughly investigated.

#### 5.4.6. Metrnβ in Hepatocellular Carcinoma (HCC)

A recent study reported a significant elevation of Metrnβ levels in HCC and identified it as a highly effective serum marker for diagnosing HCC, with a sensitivity of 90.17% [82]. The study found that serum Metrnβ levels were significantly higher in patients with AFP-negative HCC compared to those with AFP-positive HCC and metastatic carcinoma. Additionally, serum Metrnβ levels were lower in patients with advanced recurrence (2 years post-resection) than in those with early recurrence and poor survival outcomes. These findings suggest that Metrnβ may serve as a sensitive marker for identifying AFP-negative HCC patients and may be a reliable predictor of tumor progression and survival outcomes in HCC. Currently, there are no mechanistic studies in the context of Metrnβ in HCC. Given that hepatitis is a pre-stage of HCC, and that Metrnβ overexpression ameliorates hepatitis in mice by inhibiting chemokine-dependent immune cell infiltration [111], it is possible that targeting Metrnβ may be a potential therapeutic strategy for HCC, either by directly inhibiting Metrnβ or by modulating the immune response it influences.

### 5.5. Metrnβ in Allergic Diseases: Immunomodulatory Roles

Emerging evidence positions Metrnβ as a critical immunomodulator in allergic pathogenesis, with dual regulatory functions observed in both cutaneous and respiratory manifestations. Previously, Metrnβ has been shown to promote eosinophil recruitment and aggregation [2], and it was identified as a genetic locus biomarker associated with DNA methylation patterns in allergic respiratory diseases such as asthma and rhinitis [112], providing epigenetic evidence of its involvement in the molecular mechanisms underlying allergy. However, our previous research demonstrated that Metrnβ levels were significantly elevated in individuals with asthma and in mice with house dust mite (HDM)-induced allergic asthma. Treatment with recombinant mice Metrnβ protein (rmMetrnβ) resulted in a significant reduction in inflammatory cell infiltration and attenuation of allergic responses, highlighting its protective role in alleviating dendritic cell (DC)-mediated Th2 inflammation [18]. Very recently, we further found that AD patients exhibited elevated Metrnβ concentrations in lesional skin biopsies and serum, paralleled by similar elevations in DNFB-induced murine AD models. Therapeutic administration of rmMetrnβ protein significantly attenuated disease severity scores and epidermal thickening, while genetic ablation exacerbated pruritus and barrier dysfunction. Mechanistically, Metrnβ’s high-affinity binding to the KIT receptor tyrosine kinase initiates a signaling cascade that suppresses CD4+ T-cell proliferation through β-Catenin destabilization in the WNT pathway [19]. This molecular interplay downregulates IL-4/IL-13-driven inflammatory circuits, suggesting Metrnβ’s role as a checkpoint in cutaneous immune homeostasis. However, such discrepancy between researchers demands future in depth mechanistic studies.

### 5.6. Metrnβ in Infectious Diseases: Novel Biomarker and Therapeutic Target

#### 5.6.1. Metrnβ in Sepsis

Sepsis is a life-threatening condition characterized by multiple organ dysfunction resulting from a dysregulated host response to infection [113]. The immunosuppression in sepsis arises from the disruption of immune homeostasis, marked by the abnormal death of immune effector cells, excessive proliferation of immunosuppressive cells, and the release of anti-inflammatory cytokines [114]. In a mouse model of LPS-induced sepsis, Metrnβ-deficient mice exhibited more severe inflammatory symptoms and shorter survival times compared to wild-type controls, and that Metrnβ exerts protective effects in acute lung injury (ALI) by activating the SIRT1/P53/SLC7A11 signaling pathway to inhibit ferroptosis [3,84]. Similarly, Metrnβ expression was significantly elevated in the early stages of sepsis and was inversely correlated with inflammatory cytokines including TNF-α, IL-1β, IL-6, IL-8, and IL-17, with functions in promoting host immune defense by regulating Treg/Th17 immune cell balance, thereby improving survival in sepsis patients [46]. These findings suggest that Metrnβ may serve as a promising therapeutic target for sepsis by enhancing antibacterial activity in macrophages while simultaneously exerting anti-inflammatory effects.

#### 5.6.2. Metrnβ in COVID-19

The global incidence of COVID-19 has led to various complications due to frequent viral mutations, complex pathophysiology, and variable host immune responses resulted from cytokine storm syndrome and multi-organ failure [115,116]. In previous studies, we found that serum Metrnβ levels were significantly higher in COVID-19 patients linked to increased mortality, and non-survivors among critically ill patients had elevated Metrnβ concentrations relative to survivors. Additionally, Metrnβ levels were positively correlated with viral load and pro-inflammatory cytokines such as IL-10 and IL-6, indicating a potential relationship between Metrnβ and immune response dysregulation in COVID-19 [20]. However, a significant decrease in serum Metrnβ levels in patients with confirmed COVID-19 was also observed [83]. These conflicting findings may be due to variations in disease severity, differences in viral clades, as well as limited sample sizes and patient heterogeneity. Despite these inconsistencies, the current evidence suggests that Metrnβ levels are positively correlated with disease severity, making it a potential candidate for monitoring COVID-19 progression and outcomes. It is important to note that appropriate concentrations of Metrnβ may help inhibit hyperinflammatory response-mediated immunopathological damage. However, excessively high and persistently elevated levels of Metrnβ could impair antiviral immunity and promote immunopathological damage, potentially leading to secondary infections. This highlights the need for further research to define the optimal therapeutic range of Metrnβ for the effective management of COVID-19-related complications.

#### 5.6.3. Metrnβ in Chronic Obstructive Pulmonary Disease (COPD)

COPD is a prevalent lung condition characterized by chronic lung inflammation leading to airflow limitation, which can progress to pulmonary heart disease and respiratory failure [117]. In COPD exacerbation and active smokers, elevated Metrnβ levels were reported to play crucial roles in balancing inflammatory responses. The increased levels of Metrnβ in smokers during the acute phase of COPD are primarily attributed to heightened inflammation and macrophage activity. Although Metrnβ levels decrease following disease discharge, they still remain higher than healthy individuals [118], indicating that Metrnβ may play a key role during acute exacerbations of COPD, whereas the mechanisms remain to be fully addressed in the future.

### 5.7. Metrnβ in Neurological Disorders: A Protective Modulator

#### 5.7.1. Metrnβ in Cognitive Function and Aging

In a study using a D-gal-induced senescent mouse model, Metrnβ expression in the hippocampus of aging mice was significantly elevated. Knockdown of Metrnβ led to a marked reduction in BDNF levels, thereby worsening cognitive dysfunction [38]. Interestingly, Metrnβ knockdown exacerbated D-gal-induced learning deficits in senescent mice but did not noticeably affect memory function. This suggests that Metrnβ may primarily influence learning rather than memory, although further studies are needed to confirm this hypothesis. In summary, given its regulatory role in cognitive function and hippocampal BDNF levels, Metrnβ may represent a promising target for the treatment of aging-related cognitive disorders.

#### 5.7.2. Metrnβ in Inner Ear Development

Metrnβ exhibits temporally restricted expression during inner ear development [13]. In mice, Metrnβ mRNA is first detected in the inner ear at embryonic day 13.5 (E13.5), coinciding with the emergence of sensory hair cells, and intensifies by E14.5. This developmental expression aligns with critical stages of neuronal maturation and sensory epithelial organization. Notably, Metrnβ expression is transient and absent in the adult inner ear under physiological conditions, as evidenced by transcriptomic and immunohistochemical analyses. Regulatory studies in medaka fish reveal that Metrnβ is transcriptionally controlled by Pax2/8, key factors in otic vesicle development, with overexpression of Pax2/8 significantly upregulating its expression-a mechanism conserved across vertebrates. Functionally, Metrnβ demonstrates potent neuroprotective properties in a deafened guinea pig model, where intracochlear infusion of recombinant Metrnβ preserves spiral ganglion neuron (SGN) survival and maintains electrically evoked auditory brainstem response thresholds below 50 μA, even weeks post-treatment cessation. In contrast, the control group shows progressive impairment, with thresholds continuously increasing and reaching approximately 200 μA by the end of the study [13]. These effects, comparable to those of glial cell line-derived neurotrophic factor (GDNF), highlight Metrnβ’s therapeutic potential for mitigating auditory neurodegeneration secondary to hair cell loss, positioning it as a promising candidate for interventions targeting sensorineural hearing disorders.

#### 5.7.3. Metrnβ in Obstructive Sleep Apnea (OSA)

OSA is a widely studied condition, and the intermittent hypoxia associated with sleep apnea has been shown to contribute to endothelial dysfunction of the patients [119]. The pathophysiology of OSA involves intermittent hypoxia and reoxygenation, resulting in increased systemic oxidative stress and inflammation, which in turn triggers various cardiovascular events. Given the known anti-inflammatory properties of Metrnβ, it is suggested that Metrnβ may be associated with cardiovascular comorbidities in OSA patients. Recently, this hypothesis was proved as Metrnβ levels were found to be significantly lower in OSA patients compared to healthy controls and were negatively correlated with both the severity of OSA and the cardiovascular risk marker carotid intima-media thickness (CIMT); therefore, Metrnβ is a potential biomarker for identifying OSA patients at increased risk of early vascular injury [86].

## 6. Conclusions and Future Perspectives

Over the past decades, Metrnβ, a novel secreted protein with pleiotropic functions, has emerged as a cross-disciplinary research focus. This review synthesizes recent advances in Metrnβ’s roles in not only the widely studied cardiovascular-metabolic diseases and autoimmune disease, but also the emerging infectious diseases, allergic diseases, cancers, and neurological disorders, highlighting its dual potential as a therapeutic target and diagnostic biomarker for human diseases, while also addressing unresolved mechanistic ambiguities and translational barriers. Future breakthroughs should demand structural elucidation of Metrnβ via cryo-EM and tissue-specific knockout models to map receptor–ligand dynamics. Furthermore, despite preclinical consensus on Metrnβ’s therapeutic promise, clinical translation faces multidimensional challenges; we believe that integrating novel methods like multi-omics, enlarging well-designed clinical studies involving a multi-center study cohort, would help for this. In conclusion, these gaps in knowledge warrant further exploration to fully harness the mechanisms of Metrnβ’s expression profiles and modulations across diseases, as well as its potential as a biomarker and its therapeutic potential for human diseases.

## 7. Methodology

The literature selection and synthesis process for this narrative review was conducted with systematic methodological rigor to ensure transparency and reproducibility, while maintaining the conceptual depth characteristic of narrative syntheses. Initial systematic searches were performed in PubMed and Web of Science using Boolean operators combining key terms related to Metrnl, Metrnβ, cometin, subfatin, and IL-41 (e.g., “Metrnl AND biological function”, “subfatin AND disease regulation”), encompassing English-language articles published between 2000 and 2025. Following duplicate removal via EndNote X20 with cross-validation by three independent researchers, the remaining retrieved records underwent eligibility selection, with relevance to core investigative themes (discovery milestones, biological mechanisms, and disease-associated regulation) included. The study type includes but is not limited to original research articles, systematic reviews, and meta-analyses, etc. Conference abstracts were systematically excluded at this stage.

## Figures and Tables

**Table 1:** Expression and distribution patterns and potential involvement of Metrnβ in human diseases.

Types of Disease		Expression Patterns	Distribution and Cellular Sources	Potential Regulatory Roles	References
**Metabolic** **Disease**	Diabetes. Obesity.	↑/↓/unchanged ↑/↓/unchanged	Serum; adipose tissue Serum; adipose tissue	Promotes energy consumption, β-cell function and stabilizes blood glucose levels; reduces insulin resistance. Reduces lipid accumulation, regulates adipocyte differentiation, and increases thermogenesis.	[2,6,10,24,47,48,49,50,51,52,53,54,55,56,57,58,59,60,61,62,63,64,65,66,67]
**Cardiovascular Disease**	Atherosclerosis; coronary heart disease; cardiac hypertrophy and fibrosis; myocardial ischemia-reperfusion injury.	↓	Serum; heart; endothelial cell	Reduces myocardial hypertrophy, fibrosis, and inflammation; promotes myocardial remodeling and regeneration.	[29,44,47,63,65,68,69,70,71,72,73,74]
**Autoimmune Disease**	Psoriatic arthritis. Rheumatoid arthritis. Inflammatory bowel disease. Osteoarthritis. Systemic lupus erythematosus. Kawasaki disease. Spondylitis.	↑ ↑ ↑/↓ ↑ ↑ ↑ ↑/↓	Synovial fluid and tissues Synovial membranes and serum Serum; mesenteric adipose tissue Synovial fluid Serum Serum Serum	Remains to be elucidated, partially attributed to the synergistic interaction between Metrnβ with TNF and IL17A/F. Serum Metrnβ levels are closely associated with RA activity. Alleviates ulcerative colitis by promoting autophagy; ameliorates CD by promoting adipocyte function and differentiation. Ameliorates osteoarthritis by inhibiting inflammation and pyroptosis; erestores the expression of type II collagen. Positively correlated with disease activity. A diagnostic biomarker for KD; contributes to the recovery of KD by exerting anti-inflammatory effects. Decreased after inflammatory control, elevated in active disease, with mechanisms to be clarified.	
**Tumorigenesis**	Breast cancer. Cutaneous neoplasms. Malignant mesothelioma. Ovarian cancer. Colorectal cancer. Hepatocellular carcinoma.	↑ ↑/unchanged ↑ ↓ ↑/↓ ↑	Breast cancer tissue Tumor tissues, lesion area; skin Lung tissues Cancerous tissues Cancerous tissues Serum; HCC tissues	May be associated with the presence of breast cancer. A diagnostic marker for BCC, trichoblastoma, and differentiation between BCC and trichoblastoma. Metrnβ may be used as a biomarker for diagnosis of MM. May prevent cancer-related cachexia, remaining to be verified. N/A Suggests poor prognosis and postoperative recurrence in HCC; a diagnostic marker for AFP negative HCC.	[7,21,22,75,76,77,78,79,80,81,82]
**Allergic disease**	Asthma. Allergic dermatitis.	↑	Serum and lung; serum, skin and ear tissue	Alleviates DC-mediated Th2 inflammation; binds to the KIT receptor to inhibit immune cell expansion.	[18,19]
**Infectious** **Disease**	Sepsis. COVID-19. Acute lung injury.	↑ ↑/↓ ↓	Serum Serum Lung	Promotes host immune defense by regulating Treg/Th17 balance; enhances antibacterial activity of macrophages. Correlates with disease severity. Inhibits ferroptosis of alveolar epithelial cells and attenuates LPS-induced lung injury by targeting SIRT1.	[3,20,46,83,84]
**Neurological Disorder**	Inner ear development. Cognitive function and aging. Obstructive sleep apnea.	↑ ↑ ↓	Inner ear Hippocampus Serum	Supports neuronal survival by promoting neurite growth and neuroblast migration and protects spiral ganglion neurons. Alleviates aging-related cognitive dysfunction via regulating hippocampal BDNF levels. Inverse association between Metrnβ and CIMT; effective predictor of OSA.	[13,38,85,86]

Note: the ↑ indicates upregulated and ↓ indicates downregulated.

## Data Availability

No new data were created or analyzed in this study. Data sharing is not applicable to this article.

## Abbreviations

ACS	Acute coronary syndrome
AD	Allergic dermatitis
ALI	Acute lung injury
AxSpA	Ankylosing spondylitis
BDNF	Brain-derived neurotrophic factor
BIGE	Body Index of Gene Expression
CAL	Coronary artery lesion
CRP	C-reactive protein
CD	Crohn’s disease
CHD	Coronary heart disease
CIMT	Carotid intima-media thickness
CNS	Central nervous system
COPD	Chronic obstructive pulmonary disease
COVID-19	Coronavirus disease 2019
CRC	Colorectal cancer
DC	Dendritic cell
D-gal	D-galactose
ESR	Erythrocyte sedimentation rate
GD	Graves’ disease
GDNF	Glial cell line-derived neurotrophic factor
GFAP	Glial fibrillary acidic protein
HCC	Hepatocellular carcinoma
HDM	House dust mite
HFD	High-fat diet
IL-41	Interleukin-41
ILCs	Innate lymphoid cells
IVIG	Intravenous immunoglobulin
KD	Kawasaki disease
KIT	Receptor tyrosine kinase
Metrnβ	Meteorin-β
Metrnl	Meteorin-like protein
MM	Malignant mesothelioma
NGF	Nerve growth factor
OA	Osteoarthritis
OSA	Obstructive sleep apnea
PGC-1α	Peroxisome proliferator-activated receptor gamma coactivator 1-alpha
PCOS	Polycystic ovary syndrome
PPARγ	Peroxisome proliferator-activated receptor gamma
PsA	Psoriatic arthritis
RA	Rheumatoid arthritis
rmMetrnβ	Recombinant mice Metrnβ protein
RMH	Reactive mesothelial hyperplasia
RPL	Recurrent pregnancy loss
SGN	Spiral ganglion neuron
SLE	Systemic lupus erythematosus
SVZ	Subventricular zone
T2D	Type 2 diabetes
Tregs	Regulatory T cells
TrkB	Tropomyosin receptor kinase B
